# Associations of the triglyceride-glucose index and intracranial arterial stenosis with poor functional outcome after ischemic stroke: a retrospective observational study of 712 patients

**DOI:** 10.3389/fendo.2026.1949849

**Published:** 2026-10-12

**Authors:** Baoyi Liao, Yongzhe Gu, Nuo Ma, Aiping Jin, Shengqi Yao

**Affiliations:** 1 Department of Neurology, Tongren Hospital, Shanghai Jiao Tong University School of Medicine, Shanghai, China; 2 Department of Neurology, Shanghai Tenth People’s Hospital, Tongji University School of Medicine, Shanghai, China

**Keywords:** intracranial atherosclerotic stenosis, ischemic stroke, metabolic dysregulation, stroke prognosis, triglyceride-glucose index

## Abstract

**Background:**

While intracranial atherosclerosis (ICAS) and insulin resistance (IR) often co-occur in ischemic stroke, their combined prognostic impact is unclear; we hypothesized their synergy predisposes to worse short-term outcomes.

**Methods:**

We conducted a retrospective analysis of 712 consecutive patients with acute ischemic stroke. The primary outcome was poor functional status, defined as modified Rankin Scale score > 2 at 90 days. Participants were categorized according to TyG index (as a surrogate for IR) and ICAS status. Subsequent stratification based on ICAS severity (none, moderate, severe) and number of affected vessels (none, single, multiple) enabled dose-response evaluation. Subgroup analyses and interaction analyses were further performed to assess the robustness of the associations and explore potential interactive effects between TyG index and ICAS. Exploratory mediation analysis quantified the proportion of ICAS’s total statistical effect on functional outcomes that could be explained via the TyG index.

**Results:**

Univariate analysis demonstrated that both elevated TyG index (OR 1.34, 95% CI 1.01-1.79) and presence of ICAS (OR 1.74, 95% CI 1.19-2.56) were associated with poor outcomes. Although the combination of high TyG index and ICAS showed a non-significant trend (OR = 1.77, 95% CI: 0.98–3.17, p = 0.057), high TyG index significantly increased poor outcome risk in patients with severe ICAS (OR = 2.15, 95% CI: 1.13–4.09) or multiple ICAS lesions (OR = 2.11, 95% CI: 1.11–4.02). The exploratory mediation analysis showed that the mediated proportion of the TyG index in the association between ICAS and poor post-stroke functional outcome was approximately 9.0% (95% CI: −0.8% to 68.2%), and this mediated effect was not statistically significant.

**Conclusions:**

The combined assessment of the TyG index and ICAS burden shows promise for identifying stroke patients at high risk of poor prognosis.

## 1 Introduction

Intracranial atherosclerotic stenosis (ICAS), an advanced-stage manifestation of cerebral atherosclerosis, is one of the primary causes of ischemic stroke in the Chinese population (1, 2). ICAS is estimated to account for nearly half of the patients presenting with transient ischemic attack and 40% of those presenting with ischemic stroke (3). The Warfarin-Aspirin Symptomatic Intracranial Disease (WASID) study revealed that severe stenosis of the intracranial arteries was linked to a higher risk of recurrence. In cases with >70% stenosis, the annual recurrence rate of stroke exceeded 10% (4). The recurrence rate surpassed 20% in cases with vascular occlusion and >3 vascular risk factors (5). Patients with stroke due to ICAS have a particularly high residual risk despite dual antiplatelet therapy, intensive risk factor control, or endovascular treatment. The risk is especially high in patients with the highest intracranial atherosclerotic burden who face a substantial likelihood of poor stroke prognosis (6, 7). Consequently, identifying those at highest risk for poor clinical stroke-related prognosis among the ICAS population remains clinically imperative.

IR is considered a state of reduced sensitivity and responsiveness to insulin action, even for several years prior to diabetes mellitus (8). Recently, the exploration of biomarkers of IR has emerged continuously (9, 10). As a cost-effective and simple tool for assessing insulin resistance, the TyG index has recently gained attention for its association with cardiovascular disease, metabolic disorders, and poorer functional outcomes (11–13). More recently, a higher TyG index has also been reported to be strongly associated with the development of ICAS. A prior study involving 1,129 individuals diagnosed with DSA showed that higher TyG index is associated with ICAS/ECAS and further hinted that it may be more effective in the prediction of severe stenosis compared to mild/moderate stenosis (14). Besides, Yongle Wang et al. found a 1.08-fold increase in intracranial atherosclerotic stenosis burden in the fourth quartile when considering TyG as quartiles (15). However, the outcome related to the interaction or combined effect of elevated TyG index with and without ICAS in patients with symptomatic cerebrovascular disease is uncertain.

In view of the established diagnostic value of the TyG index for insulin resistance and these documented associations, we hypothesized a strong relationship between the TyG index and cerebrovascular disease. To offer additional evidence of the significance of TyG index for cerebral atherosclerotic stenosis and to improve our understanding of the pathophysiologic mechanisms in stroke patients, We therefore conducted this study to explore whether combined presence of the TyG index and the ICAS burden can be used to predict the short-term prognosis of stroke.

## 2 Methods

### 2.1 Study population

The study cohort consisted of patients diagnosed with acute ischemic stroke who underwent CTA/MRA at Shanghai Tenth People’s Hospital between August 2023 and August 2024. The diagnosis of ischemic stroke was determined by assessing the patient’s clinical features (16) and examining neuroimaging data acquired from CT or magnetic resonance imaging (MRI) scans of the brain (17).

The exclusion criteria were as follows: (1) transient ischemic attack (TIA); (2) treatment with recombinant tissue plasminogen activator (rt-PA) or endovascular thrombectomy; (3) presence of severe comorbidities, including active infections, malignant tumors, hematologic or autoimmune diseases, or chronic liver or renal insufficiency; (4) pre-stroke modified Rankin Scale (mRS) score ≥ 2; (5) missing data on triglycerides or fasting plasma glucose; (6) missing intracranial arterial imaging data; and (7) non-atherosclerotic intracranial stenosis. **Figure 1** shows the flowchart for patient screening and exclusion. In total, 712 patients were included in the final analysis.

**Figure 1:**
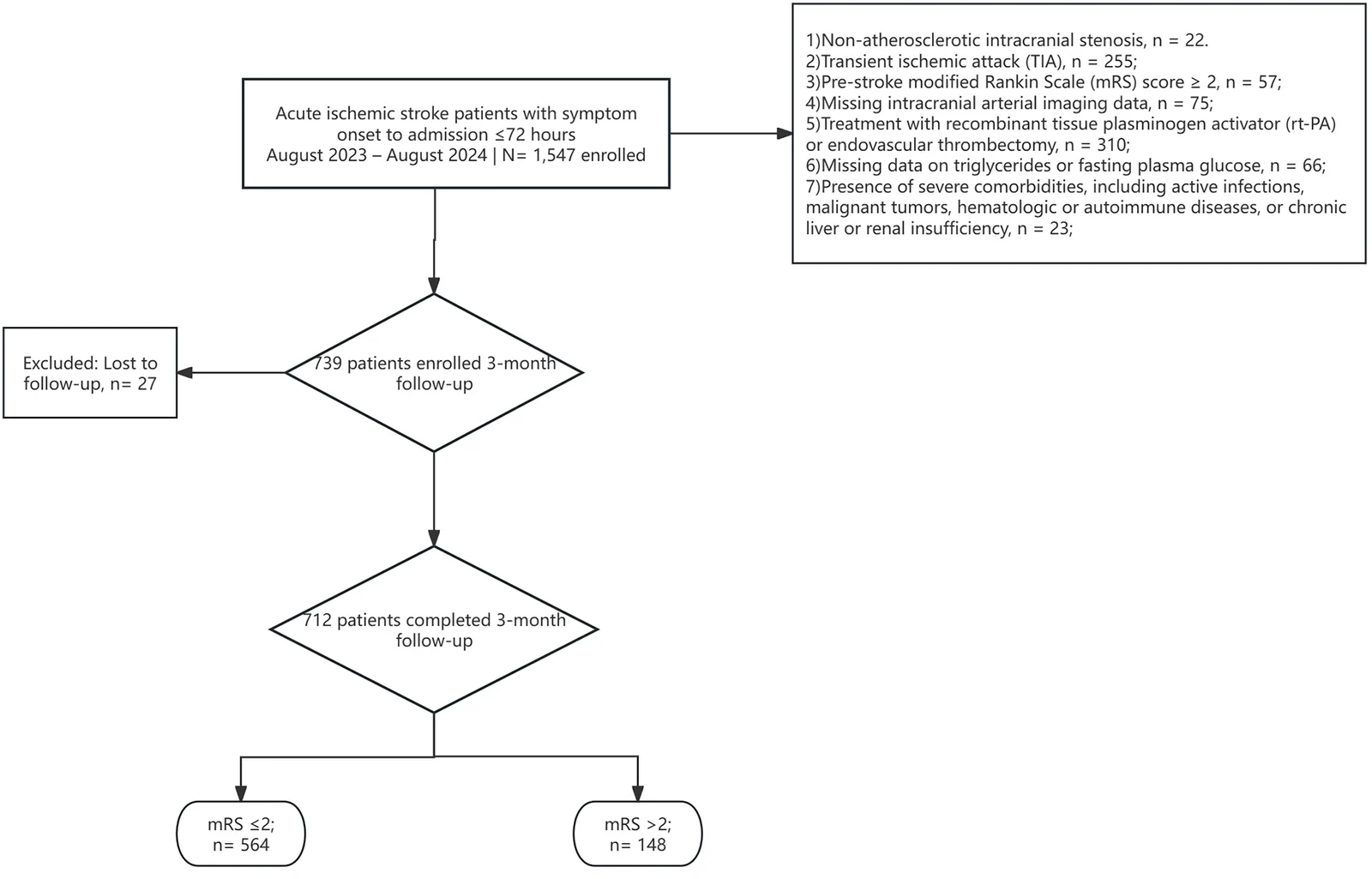
Flow chart of patient recruitment.

### 2.2 CTA/MRA acquisition and image analysis

CTA/MRA were employed to evaluate the number and degree of stenotic vessels. Two imaging physicians, blinded to the patients’ clinical profiles, independently evaluated all images, and any disparities in their assessments were resolved through negotiation.

The following vessels were evaluated using the Warfarin and Aspirin for Symptomatic Intracranial Arterial Stenosis (WASID) method (18): the M1-M2 segment of the middle cerebral artery, the A1-A2 segment of the anterior cerebral artery, the P1-P2 segment of the posterior cerebral artery, the V4 segment of the vertebral artery, the C6-C7 segments of the internal carotid artery, and the basilar artery. ICAS was defined as the presence of atherosclerotic stenosis or occlusion in a single major intracranial artery exceeding 50%. Patients with ≥ 70% intracranial stenosis or major vessel occlusion were considered to have severe stenosis. ICAS involving ≥2 segments was defined as multi-segment ICAS.

### 2.3 Clinical data collection

All required information was retrieved from the patients’ computerized medical records. Data collected included general information (age, gender, BMI, smoking and drinking status, admission SBP and DBP), medical history (hypertension, diabetes, atrial fibrillation, stroke, coronary heart disease), and laboratory results (TC, TG, HDL-C, LDL-C, FBG). Blood samples were collected after a minimum 8-hour fast for laboratory analysis, which was completed within 24 hours after hospital admission. Stroke severity was assessed on admission by a qualified neurologist using the National Institutes of Health Stroke Scale (NIHSS) (19). The TyG index was calculated as ln[TG(mg/dL) × FBG (mg/dL)/2] (20).

### 2.4 Statistical analysis

Continuous variables were expressed as means (SD) and compared using t-tests, while categorical variables were expressed as counts and proportions and compared using chi-square tests. Univariate and multivariable logistic regression models were employed to analyze the relationships between TyG index, ICAS status, and ischemic stroke poor outcome. Model 1 presented unadjusted analyses, while Model 2 incorporated adjustments for the predetermined covariates (see **Table 1** footnote).

**Table 1:** Association of the TyG index with poor prognosis after ischemic stroke: a multivariable logistic regression analysis.

Characteristic	Model 1					Model 2		
	N	Event N	OR	95% CI	P-value	OR	95% CI	P-value
TyG (continuous)	712	148	1.34	1.01, 1.79	0.043	1.46	1.03, 2.08	0.035
TyG (standardized)	712	148	1.20	1.01, 1.44	0.043	1.27	1.02, 1.57	0.035
TyG								
Q1	178	33	---	---	---	---	---	---
Q2	178	39	1.23	0.73, 2.07	0.429	1.24	0.69, 2.22	0.479
Q3	178	29	0.86	0.49, 1.48	0.576	0.88	0.46, 1.66	0.689
Q4	178	47	1.58	0.95, 2.61	0.077	1.62	0.87, 3.00	0.126
P for trend					0.187			0.224

Model 1: No covariates were adjusted.

Model 2: Adjusted for Sex, Age, BMI, SBP at admission, DBP at admission, Current cigarette smoking, Current alcohol drinking, Hypertension, Stroke, Atrial fibrillation, Antiplatelet therapy, Coronary heart disease, Statin therapy, HDL, and LDL.

Odds ratios (ORs) with 95% confidence intervals (CIs) were estimated. The correlation heatmap presents the relationships among various clinical and demographic variables (**Figure 2**).

**Figure 2:**
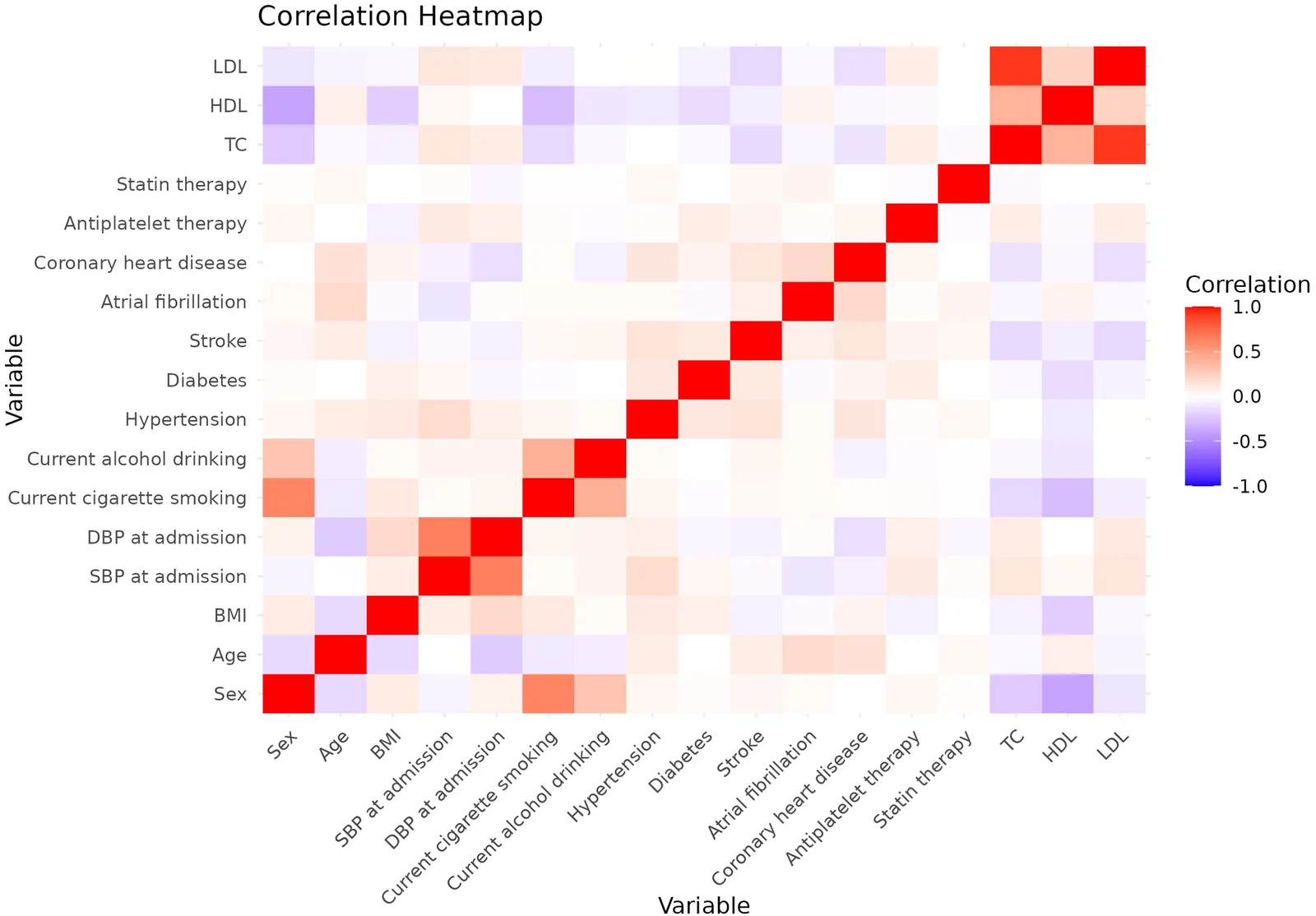
Correlation heatmap. The correlation heatmap presents the relationships between various clinical and demographic variables. Each cell within the grid represents the correlation coefficient between the variables, ranging from -1 to 1, with 1 indicating a perfect positive correlation, -1 a perfect negative correlation, and values close to 0 suggesting no correlation.

Possible nonlinear relationships between the change in TyG and stroke poor outcome were examined by a logistic regression model with restricted cubic splines (RCS). We conducted RCS with 4 knots at the 5th, 35th, 65th, and 95th percentiles to flexibly model the association. Additionally, the exploratory mediation analysis evaluated the magnitude of potential mediating effects between variables. A Quasi-Bayesian approach was employed to estimate the mediation effects, with 100 simulations performed to ensure robustness of the results. The exploratory mediation analysis encompassed the evaluation of both the total, indirect, and direct effects of ICAS on stroke poor outcome, stating confidence intervals and statistical significance for each effect. Notably, given the retrospective single-time-point observational design, this analysis represents purely statistical decomposition rather than proof of biological causal pathways.

Subgroup analyses were conducted based on Sex, Age, BMI, Hypertension, and Diabetes to explore potential effect modifiers and assess the robustness of the main findings across different patient populations. Interaction analyses were further performed to evaluate multiplicative and additive interactions between TyG index and ICAS status.

To mitigate the potential bias caused by missing values in data analysis, we assumed that missing data were missing at random. We utilized the multiple imputation by chained equations method based on baseline characteristics to establish a complete dataset. The statistical analyses were conducted using R (version 4.2.2; R Foundation for Statistical Computing, Vienna, Austria). Two-tailed p < 0.05 was considered statistically significant.

## 3 Results

### 3.1 Baseline characteristics

The fundamental characteristics of the participants are displayed in **Table 2**. Among the participants, the average age was 67 ± 10 years, and 49.6% were male. The mean TyG index value was 8.70 ± 0.62. Individuals with a higher TyG index and ICAS burden exhibited elevated BMI, SBP, DBP, FBG, TC, TG, LDL-C levels, and reduced HDL-C levels. Patients with higher TyG index and ICAS burden were more likely to experience atherosclerotic stroke, with a lower incidence of cardioembolic stroke. In contrast, no significant differences were found in sex distribution, admission NIHSS score, cigarette smoking, alcohol consumption, atrial fibrillation, coronary heart disease, or antiplatelet and statin therapies between different groups.

**Table 2:** Baseline characteristics.

Characteristic	Overall, N = 712	Lower TyG without ICAS N = 183	Higher TyG without ICAS N = 122	Lower TyG with ICAS N = 177	Higher TyG with ICAS N = 230	P-value
Male	353 (49.6%)	89 (48.6%)	67 (54.9%)	83 (46.9%)	114 (49.6%)	0.579
Age	67 ± 10	68 ± 10	64 ± 9	70 ± 10	67 ± 10	<0.001
BMI	24.4 ± 3.4	23.6 ± 3.5	25.3 ± 3.4	24.2 ± 3.3	24.6 ± 3.4	<0.001
SBP at admission	149 ± 21	146 ± 20	151 ± 20	146 ± 19	152 ± 22	<0.001
DBP at admission	88 ± 13	87 ± 12	92 ± 13	85 ± 13	88 ± 14	<0.001
Admission NIHSS score	2.50 (1.00, 4.00)	2.00 (1.00, 4.00)	3.00 (1.00, 5.00)	3.00 (1.00, 5.00)	2.00 (1.00, 4.00)	0.296
Current cigarette smoking	201 (28.2%)	51 (27.9%)	35 (28.7%)	55 (31.1%)	60 (26.1%)	0.741
Current alcohol drinking	73 (10.3%)	15 (8.2%)	17 (13.9%)	19 (10.7%)	22 (9.6%)	0.423
Hypertension	529 (74.3%)	120 (65.6%)	88 (72.1%)	129 (72.9%)	192 (83.5%)	<0.001
Diabetes	350 (49.2%)	46 (25.1%)	85 (69.7%)	65 (36.7%)	154 (67.0%)	<0.001
Stroke	162 (22.8%)	30 (16.4%)	23 (18.9%)	46 (26.0%)	63 (27.4%)	0.028
Atrial fibrillation	61 (8.6%)	18 (9.8%)	9 (7.4%)	22 (12.4%)	12 (5.2%)	0.064
Coronary heart disease	113 (15.9%)	24 (13.1%)	17 (13.9%)	33 (18.6%)	39 (17.0%)	0.456
TG	1.43 ± 0.75	0.98 ± 0.30	1.85 ± 0.69	1.00 ± 0.26	1.90 ± 0.87	<0.001
GLU	6.21 ± 2.49	4.95 ± 0.92	6.96 ± 2.47	4.96 ± 0.90	7.79 ± 3.07	<0.001
TyG	8.70 ± 0.62	8.20 ± 0.31	9.12 ± 0.39	8.23 ± 0.31	9.22 ± 0.47	<0.001
TC	4.33 ± 1.07	4.14 ± 1.02	4.53 ± 0.95	4.11 ± 1.06	4.54 ± 1.13	<0.001
HDL	1.09 ± 0.26	1.18 ± 0.29	1.05 ± 0.25	1.11 ± 0.25	1.01 ± 0.22	<0.001
LDL	2.71 ± 0.92	2.59 ± 0.88	2.81 ± 0.84	2.61 ± 0.94	2.83 ± 0.96	0.013
Toast						<0.001
Lacunar	320 (44.9%)	110 (60.1%)	89 (73.0%)	56 (31.6%)	65 (28.3%)	
Large-artery atherosclerosis	272 (38.2%)	19 (10.4%)	12 (9.8%)	95 (53.7%)	146 (63.5%)	
Cardioembolism	56 (7.9%)	21 (11.5%)	11 (9.0%)	15 (8.5%)	9 (3.9%)	
Other	64 (9.0%)	33 (18.0%)	10 (8.2%)	11 (6.2%)	10 (4.3%)	
Antiplatelet therapy						0.970
Single antiplatelet therapy	280 (39.3%)	70 (38.3%)	50 (41.0%)	69 (39.0%)	91 (39.6%)	
Dual antiplatelet therapy	432 (60.7%)	113 (61.7%)	72 (59.0%)	108 (61.0%)	139 (60.4%)	
Statin therapy	689 (96.8%)	176 (96.2%)	118 (96.7%)	174 (98.3%)	221 (96.1%)	0.578
ICAS	407 (57.2%)	0 (0.0%)	0 (0.0%)	177 (100.0%)	230 (100.0%)	<0.001
Severe stenosis of ICAS	231 (32.4%)	0 (0.0%)	0 (0.0%)	93 (52.5%)	138 (60.0%)	<0.001
Multi-Stenosis of ICAS	209 (29.4%)	0 (0.0%)	0 (0.0%)	84 (47.5%)	125 (54.3%)	<0.001

### 3.2 Univariate analysis of predictors for poor stroke outcome

**Figure 3** demonstrates that poor functional prognosis after ischemic stroke was significantly associated with age (OR = 1.04, 95% CI: 1.01–1.06), admission systolic blood pressure (OR = 1.01, 95% CI: 1.00–1.02), previous stroke (OR = 1.67, 95% CI: 1.11–2.50), the TyG index (OR = 1.34, 95% CI: 1.01–1.79), and ICAS (OR = 1.74, 95% CI: 1.19–2.56). Conversely, BMI was identified as a potential protective factor (OR = 0.94, 95% CI: 0.89–1.00). In the overall population, the TyG index exhibited a linear relationship with adverse outcomes (p for nonlinearity = 0.990). A consistent linear dose-response relationship was observed in both patients with and without ICAS (p for nonlinearity = 0.850 and 0.294, respectively) (**Figure 4**).

**Figure 3:**
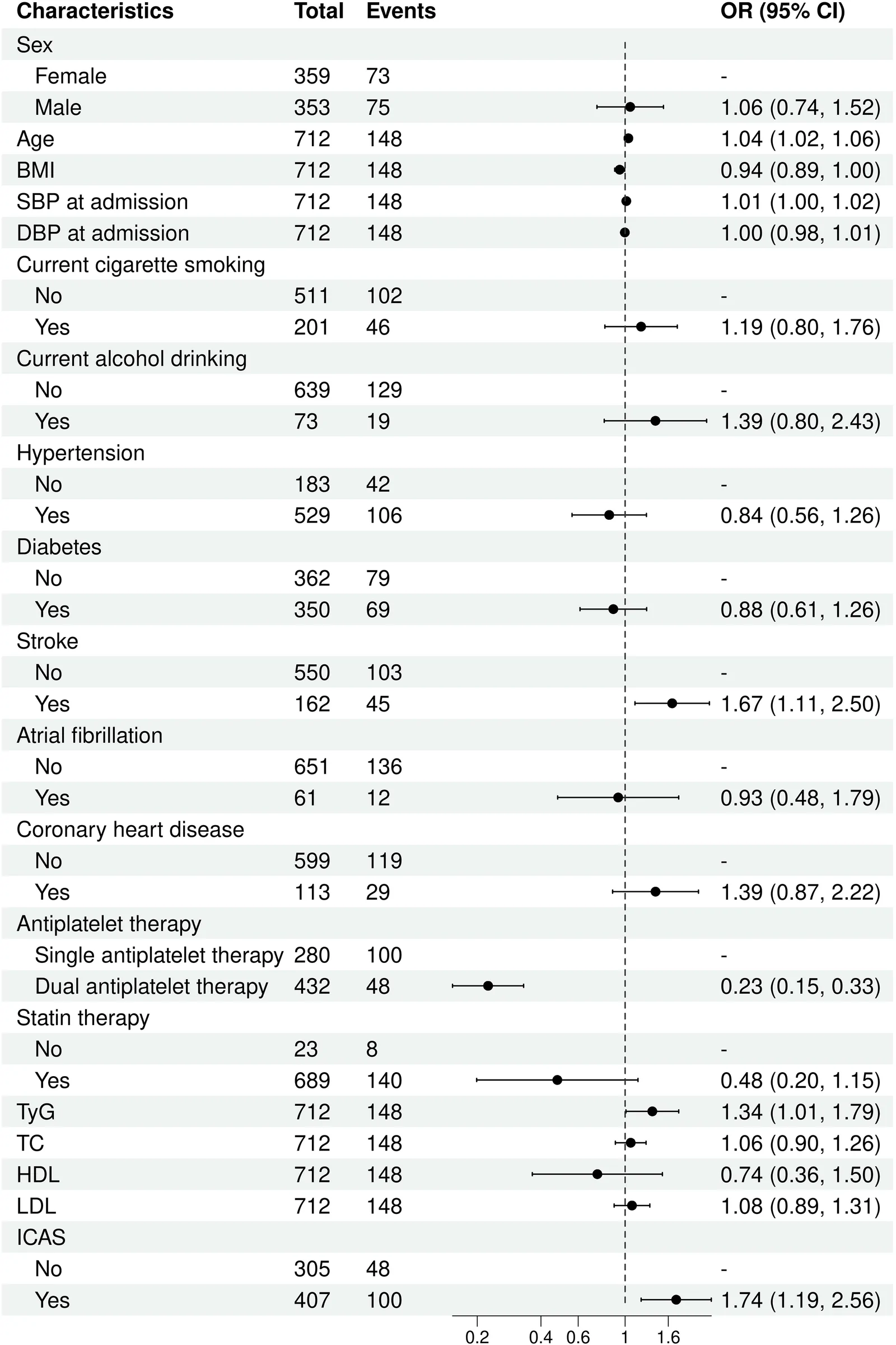
Univariate analysis of predictors for poor stroke outcome.

**Figure 4:**
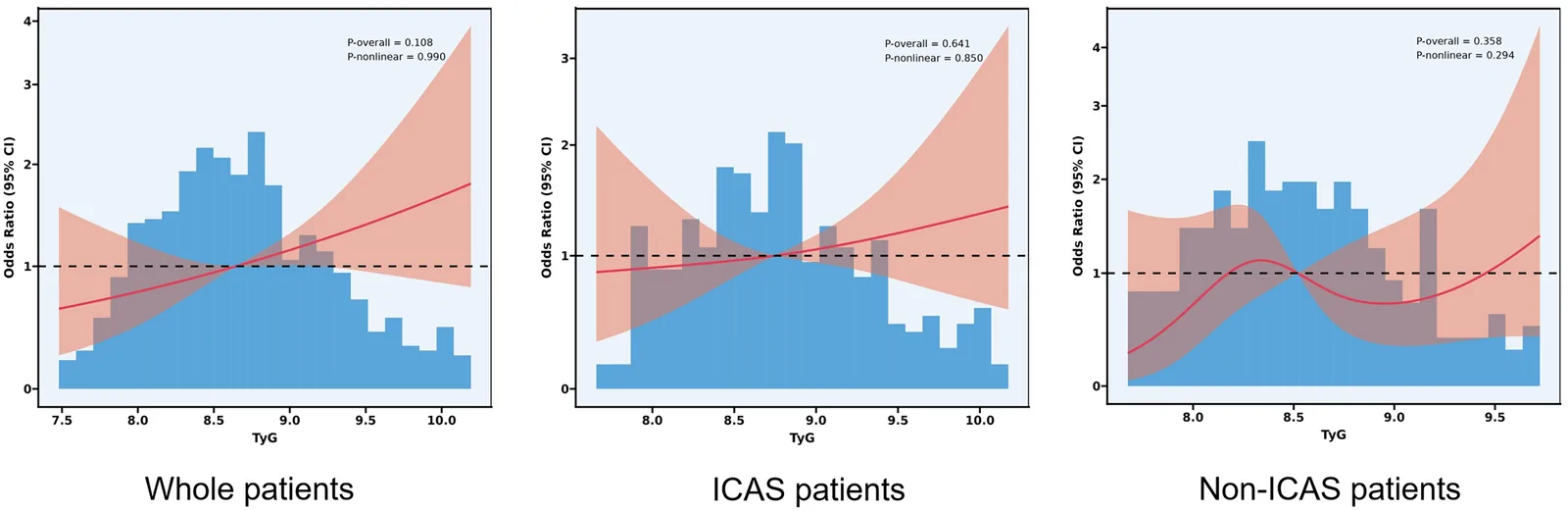
Restricted cubic spline plots of the association between TyG index and poor prognosis of stroke. Adjusted for Sex, Age, BMI, SBP at admission, DBP at admission, Current cigarette smoking, Current alcohol drinking, Hypertension, Stroke, Atrial fibrillation, Antiplatelet therapy, Coronary heart disease, Statin therapy, HDL, and LDL.

### 3.3 Association of the TyG index with poor prognosis after ischemic stroke: a multivariable logistic regression analysis

As shown in **Table 1**, in the unadjusted model (Model 1), each unit increase in the TyG index was associated with a 34% higher risk of poor post-stroke functional outcomes (OR = 1.34, 95% CI: 1.01–1.79, p = 0.043). This association remained significant after adjustment for demographic and clinical risk factors in Model 2 (OR = 1.46, 95% CI: 1.03–2.08, p = 0.035). Similarly, each standard deviation increase in the standardized TyG index was associated with increased odds of the outcome across all models (Model 1: OR = 1.20, 95% CI: 1.01–1.44, p = 0.043; Model 2: OR = 1.27, 95% CI: 1.02–1.57, p = 0.035). In the quartile-based analysis, the association for the highest quartile (Q4) did not reach statistical significance in the unadjusted model (OR = 1.58, 95% CI: 0.95–2.61, p = 0.077) or after covariate adjustment (OR = 1.62, 95% CI: 0.87–3.00, p = 0.126). Accordingly, the p for trend across TyG quartiles did not reach statistical significance in either the unadjusted model (p for trend = 0.187) or the fully adjusted model (p for trend = 0.224).

### 3.4 Effect of TyG, ICAS burden on poor clinical stroke-related prognosis

As summarized in **Table 3**, patients with concomitant elevation in the TyG index and increased ICAS burden showed a 1.72-fold increased risk of poor stroke outcome in the unadjusted model (OR = 1.72, 95% CI: 1.05–2.81, p = 0.031). This association was attenuated and no longer statistically significant in the fully adjusted model (OR = 1.77, 95% CI: 0.98–3.17, p = 0.057). A significant dose-response trend was observed across the six predefined subgroups, stratified by ascending TyG index and ICAS burden (all p for trend < 0.05). In the fully adjusted analysis, patients with severe ICAS and high TyG index had a significantly elevated risk compared to the reference group (adjusted OR = 2.15, 95% CI: 1.13–4.09). Further analysis by number of stenotic segments indicated that poor prognosis was primarily evident in patients with multiple ICAS stenoses together with an elevated TyG index (adjusted OR = 2.11, 95% CI: 1.11–4.02).

**Table 3:** Effect of TyG, ICAS burden on stroke poor clinical prognosis.

Characteristic	N	Event N	Model 1			Model 2			
			OR	95% CI	*P*-value	OR	95% CI	*P*-value	
Synergistic effect of TyG index combined with ICAS on poor functional prognosis									
Lower TyG without ICAS	183	30	—	—		—	—		
Higher TyG without ICAS	122	18	0.88	0.47, 1.67	0.700	0.97	0.48, 1.96	0.923	
Lower TyG with ICAS	177	42	1.59	0.94, 2.68	0.083	1.56	0.88, 2.78	0.131	
Higher TyG with ICAS	230	58	1.72	1.05, 2.81	0.031	1.77	0.98, 3.17	0.057	
P for trend					0.009			0.021	
Risk of poor functional prognosis in subgroup with TyG index combined with ICAS lesion segments​									
Lower TyG without ICAS	183	30	—	—		—	—		
Higher TyG without ICAS	122	18	0.88	0.47, 1.67	0.700	0.95	0.47, 1.92	0.888	
Lower TyG+1 ICAS segment	93	17	1.14	0.59, 2.20	0.694	1.23	0.60, 2.53	0.568	
Higher TyG+1 ICAS segment	105	20	1.20	0.64, 2.24	0.567	1.34	0.65, 2.74	0.427	
Lower TyG+ multiple ICAS segment	84	25	2.16	1.17, 3.98	0.013	1.92	0.98, 3.78	0.058	
Higher TyG+ multiple ICAS segment	125	38	2.23	1.29, 3.85	0.004	2.11	1.11, 4.02	0.023	
P for trend					<0.001			0.004	
Risk of poor functional prognosis in subgroup with TyG index combined with ICAS stenosis severity									
Lower TyG without ICAS	183	30	—	—		—	—		
Higher TyG without ICAS	122	18	0.88	0.47, 1.67	0.700	0.96	0.47, 1.95	0.916	
Lower TyG + moderate stenosis	84	16	1.20	0.61, 2.35	0.594	1.22	0.58, 2.55	0.597	
Higher TyG + moderate stenosis	92	20	1.42	0.75, 2.66	0.280	1.31	0.63, 2.70	0.470	
Lower TyG + severe stenosis	93	26	1.98	1.09, 3.60	0.025	1.93	0.99, 3.76	0.055	
Higher TyG + severe stenosis	138	38	1.94	1.13, 3.33	0.017	2.15	1.13, 4.09	0.019	
P for trend					0.001			0.003	

OR, Odds Ratio; CI, Confidence Interval.

Model 1: No covariates were adjusted.

Model 2: Adjusted for Sex, Age, BMI, SBP at admission, DBP at admission, Current cigarette smoking, Current alcohol drinking, Hypertension, Stroke, Atrial fibrillation, Antiplatelet therapy, Coronary heart disease, Statin therapy, HDL, and LDL.

### 3.5 Exploratory mediation analysis

Mediation analysis (**Table 4**) demonstrated a significant total effect of ICAS on poor stroke outcome (coefficient = 0.07392, 95% CI: 0.00935–0.12620, p = 0.020). The analysis further identified a significant indirect effect mediated by the TyG index (coefficient = 0.00667, 95% CI: 0.00012–0.01419, p = 0.040). In contrast, the direct effect of ICAS did not reach statistical significance (coefficient = 0.06725, 95% CI: −0.00035–0.12302, p = 0.060). The proportion of mediation attributable to TyG was 9.0% (95% CI: −0.8–68.2), with the pathway illustrated in **Figure 5**.

**Table 4:** Causal mediation analysis of ICAS and stroke poor outcome: the role of TyG as a mediator.

Independent variable	Mediator	Total effect		Indirect effect		Direct effect		Proportion mediated (95% CI)
		Coefficient (95% CI)	P value	Coefficient (95% CI)	P value	Coefficient (95% CI)	P value	
ICAS	TyG	0.07392 (0.00935, 0.12620)	0.020	0.00667 (0.00012, 0.01419)	0.040	0.06725 (-0.00035, 0.12302)	0.060	9.0 (-0.8, 68.2)

The mediation analyses were adjusted for Sex, Age, BMI, SBP.at.admission, DBP.at.admission, Current.cigarette.smoking, Current.alcohol.drinking, Hypertension, Stroke, Atrial.fibrillation, Coronary.heart.disease, Antiplatelet.therapy, Statin.therapy, LDL and HDL.

**Figure 5:**
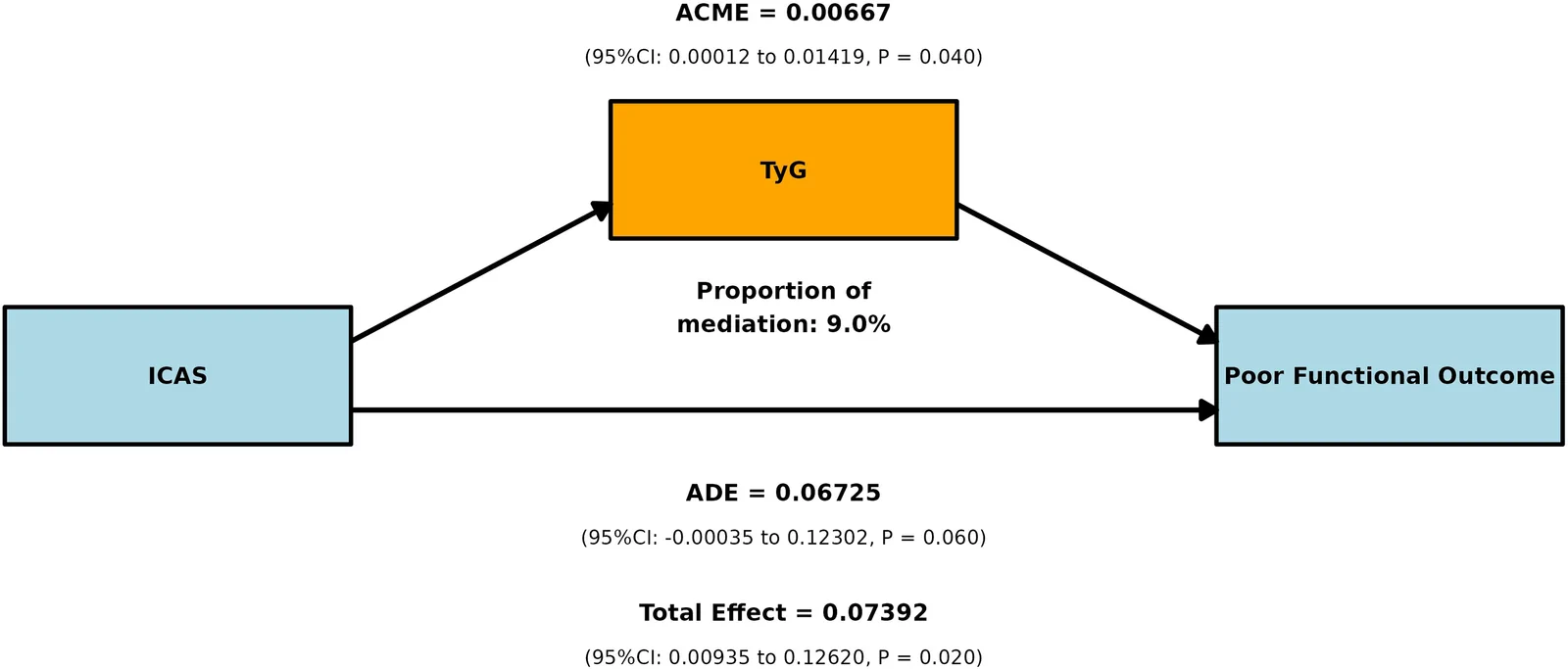
Path diagram for statistical decomposition of associations among ICAS, TyG index, and poor functional outcome. ACME, Average Causal Mediation Effect; ADE, Average Direct Effect.

### 3.6 Subgroup analysis

In the overall cohort of 712 patients, a higher TyG index was significantly associated with an increased risk of poor functional outcome, with an adjusted odds ratio (OR) of 1.46 (95% CI: 1.03–2.08, p = 0.035). Subgroup analyses revealed no significant effect modification by sex (p for interaction = 0.281), BMI group (p for interaction = 0.851), hypertension status (p for interaction = 0.985), or diabetes status (p for interaction = 0.694). A trend toward effect modification by age was observed (p for interaction = 0.059), with a significant association between TyG and outcome in patients aged ≥65 years (OR = 1.80, 95% CI: 1.17–2.77, p = 0.008) but not in those < 65 years (OR = 0.86, 95% CI: 0.42–1.77, p = 0.686) (**Figure 6**).

**Figure 6:**
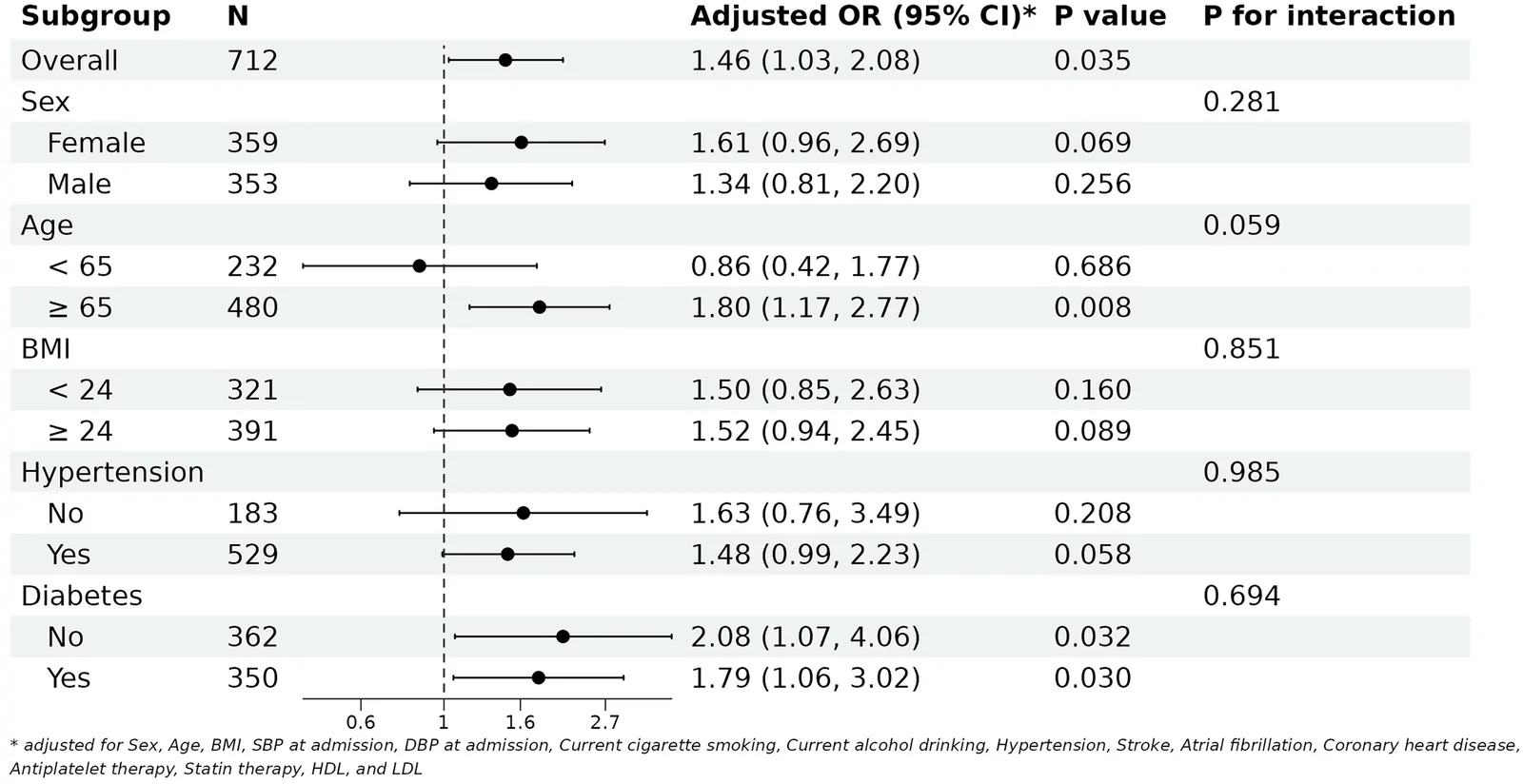
Subgroup analyses of the association between TyG index and poor functional outcome.

### 3.7 Interaction effect analysis

In the unadjusted Model 1, no significant multiplicative interaction was observed (OR = 1.228, 95% CI 0.562–2.685, p = 0.607). After adjusting for prespecified confounders in Model 2, the multiplicative interaction remained non-significant (OR = 1.173, 95% CI 0.503–2.737, p = 0.713). For additive interaction, all indicators including RERI, AP and SI had 95% confidence intervals covering the null value in the fully-adjusted model (RERI = 0.241, 95% CI −0.796–1.278; AP = 0.136, 95% CI −0.445–0.718; SI = 1.458, 95% CI −1.433–4.349), suggesting no statistically significant additive interaction. The interaction analysis results are presented in **Table 5**.

**Table 5:** Multiplicative and additive interaction of TyG index and ICAS for poor post-stroke functional outcome.

Interactive items	Interactive effects(95% CI)					
	Model 1			Model 2		
Additive effects	Estimates	95% CI	P for interaction	Estimates	95% CI	P for interaction
RERI	0.250	−0.669–1.170	---	0.241	−0.796–1.278	---
AP	0.146	−0.388–0.679	---	0.136	−0.445–0.718	---
SI	1.534	−1.543–4.610	---	1.458	−1.433–4.349	---
Multiplicative effects	1.228	0.562,2.685	0.607	1.173	0.503,2.737	0.713

Model 1: No covariates were adjusted.

Model 2: Adjusted for Sex, Age, BMI, SBP at admission, DBP at admission, Current cigarette smoking, Current alcohol drinking, Hypertension, Stroke, Atrial fibrillation, Antiplatelet therapy, Coronary heart disease, Statin therapy, HDL, and LDL.

AP, attributable proportion due to interaction; CI,confidence interval; RERI, relative excess risk due to interaction; SI, synergy index.

### 3.8 The incremental prognostic ability of the TyG index with ICAS

After adding TyG and ICAS indicators to Model 2, the new model demonstrated a statistically significant improvement in the integrated discrimination improvement (IDI) index. However, no significant enhancements were observed in the overall predictive discrimination, as measured by the C-statistic, or in reclassification ability, assessed by categorical, event, and non-event net reclassification improvement (NRI) (**Table 6**).

**Table 6:** The incremental prognostic ability of the TyG index with ICAS.

Variable	Metric	Estimate	95% CI	P-value
Model 2	C-statistic	0.762	(0.721-0.804)	0.281
Model 2+ TyG and ICAS		0.770	(0.730-0.811)	
Model 2	IDI	Reference	Reference	0.024
Model 2 + TyG and ICAS		0.014	(0.003 - 0.039)	
Model 2	Categorical-NRI	Reference	Reference	0.616
Model 2 + TyG and ICAS		0.035	(-0.047-0.098)	
Model 2	Event-NRI	Reference	Reference	0.540
Model 2+ TyG and ICAS		0.041	(-0.040-0.084)	
Model 2	Non-Event NRI	Reference	Reference	0.840
Model 2+ TyG and ICAS		-0.005	(-0.028-0.034)	

TyG: triglyceride-glucose; ICAS: intracranial arterial stenosis; CI: confidence interval; IDI: integrated discrimination improvement; NRI: net reclassification index.

Model 2: Adjusted for Sex, Age, BMI, SBP at admission, DBP at admission, Current cigarette smoking, Current alcohol drinking, Hypertension, Stroke, Atrial fibrillation, Antiplatelet therapy, Coronary heart disease, Statin therapy, HDL, and LDL.

## 4 Discussion

This study demonstrated that patients exhibiting both elevated TyG index and increased ICAS burden tended to have higher risk of poor functional outcome at 90 days, with significant associations observed in subgroups of severe and multi-vessel ICAS. This combined approach may help to identify patients at high risk of poor stroke outcomes, potentially leading to more targeted risk-management strategies and appropriate medical decisions for patients with acute ischemic stroke. Notably, these findings are based on a cohort that excluded patients receiving guideline-indicated reperfusion therapies (intravenous thrombolysis or endovascular thrombectomy). We did not include these patients for the following considerations. While including reperfusion-treated patients would improve external validity for real-world patients with acute ischemic stroke, several practical study-related factors guided this exclusion.

First, reperfusion decisions for patients with mild-severity stroke are highly variable among clinicians, especially for those with large-vessel occlusion and minor symptoms. Including such patients would introduce substantial sample heterogeneity, as physician-related treatment preference itself represents a source of selection bias. Second, reperfusion therapy directly alters vascular recanalization status and reshapes functional recovery trajectories. If reperfusion-treated patients had been included, treatment-related therapeutic effects might dilute or obscure the intrinsic prognostic associations of baseline metabolic and atherosclerotic markers that this study aimed to evaluate. Third, eligibility for thrombolysis or thrombectomy is strictly time-window dependent; onset-to-admission time is itself a strong prognostic factor that could not be fully adjusted for within our dataset. Fourth, reperfusion-related adverse events, including hemorrhagic transformation and reperfusion-triggered inflammation, may confound the associations among the TyG index, intracranial atherosclerotic burden, and post-stroke functional outcome.

Taken together, we excluded patients receiving reperfusion therapies to minimize these multiple potential confounders, allowing a clearer evaluation of the baseline prognostic roles of the TyG index and intracranial atherosclerotic burden within this mild-stroke subgroup. Nevertheless, this exclusion inevitably limits the generalizability of our findings. Our results cannot be directly extrapolated to patients who have undergone intravenous thrombolysis or endovascular thrombectomy, and future validation in cohorts including reperfusion-treated acute ischemic-stroke patients is needed.

Prior epidemiological studies have linked elevated TyG index to increased risk of multiple cardiovascular diseases and metabolic abnormalities. This association could potentially provide insight into the mechanisms underlying the onset and prognosis of stroke (21–30). Miao, Mengyuan et al. conducted a study using carotid ultrasonography data to evaluate aberrant carotid intima-media thickness (cIMT) in patients and found that increased TyG index was linked to carotid atherosclerosis in IS patients, suggesting that TyG may serve as a valuable marker for atherosclerosis (21). A large nationally representative cohort study that included 12,326 clinically asymptomatic Korean adults demonstrated that TyG index is independently associated with coronary artery calcification progression (22). Similarly, TyG index can also be used for predicting the onset of nonalcoholic fatty liver (23), metabolic disorders (24), atrial fibrillation (25, 26), hyperuricemia (27), hypertension (28, 29), and chronic kidney disease (30).

We performed mediation-based statistical decomposition to explore the inter-relationships among ICAS, TyG index, and post-stroke functional prognosis. The statistical indirect component involving the TyG index accounted for approximately 9.0% of the total association between ICAS and poor clinical prognosis, although the direct effect was non-significant. Nevertheless, the broad confidence interval of this proportion reflects substantial uncertainty, which may stem from sample-size constraints or unmeasured confounders. These observations underscore the clinical relevance of metabolic factors within the ICAS-prognosis association.

While reversing the mediation model (TyG as exposure, ICAS as mediator) would be more pathophysiologically plausible, valid implementation requires pre-stroke TyG measurements to establish proper temporal ordering. Such premorbid metabolic data are not available in our cohort, where TyG was only assessed at acute-stroke admission. Accordingly, we could not reliably fit this alternative mediation structure. For this reason, we retained the original statistical decomposition and explicitly note that these outputs represent purely observational statistical partitioning and cannot prove underlying biological pathways. Future prospective longitudinal studies with serial pre-stroke metabolic and vascular assessments are needed to disentangle these directional inter-relationships.

Our subgroup analysis showed that elevated TyG index was significantly associated with poor functional outcome only in older patients (≥65 years, OR = 1.80, 95% CI: 1.17–2.77), but not in younger individuals (< 65 years, OR = 0.86, 95% CI: 0.42–1.77). Several biological and clinical mechanisms may explain this age-dependent prognostic effect.

First, older stroke patients generally have a higher prevalence and more severe insulin resistance, which is precisely reflected by the TyG index. Aging exacerbates peripheral insulin resistance, promotes vascular endothelial dysfunction, and accelerates intracranial atherosclerotic progression, thereby amplifying the predictive value of the TyG index for adverse vascular outcomes. Second, age-related vascular structural and functional deterioration, including increased arterial stiffness, impaired endothelial repair capacity, and chronic low-grade vascular inflammation, renders older vessels more vulnerable to metabolic stress. Therefore, the metabolic abnormality captured by the TyG index exerts a more pronounced prognostic impact in the elderly population. Third, younger stroke patients typically have more heterogeneous stroke etiologies rather than chronic metabolic atherosclerosis, which may dilute the prognostic significance of the TyG index in younger subgroups. Notably, the interaction p-value was marginally nonsignificant (0.059). Therefore, this age-modifying effect should be interpreted cautiously as hypothesis-generating and requires further large-scale prospective cohorts for validation.

Our study showed that the adverse prognostic effect of elevated TyG index was only prominent among patients with severe or multiple intracranial atherosclerotic lesions. These findings suggest that metabolic stress becomes harmful only once cerebrovascular reserve drops below a critical threshold, which may explain why the prognostic effect of the TyG index was restricted to patients with severe or multi-vessel ICAS.

Insulin resistance is closely associated with reduced cerebral arterial blood flow and impaired cortical perfusion, which gradually consumes intrinsic cerebrovascular compensatory capacity (31). In intracranial atherosclerotic disease, collateral circulation serves as a decisive protective factor against ischemic injury, and its protective buffer effect is substantially weakened in patients with severe stenosis or multi-vessel lesions (32). Consistently, impaired distal perfusion independently predicts aggravated ischemic injury and poor clinical outcomes in symptomatic ICAS patients (33).

Mechanistically, elevated TyG index indicates synergistic metabolic stress of hyperglycemia and hypertriglyceridemia, which induces oxidative stress, advanced glycation end products (AGE) deposition, and excessive release of pro-inflammatory cytokines, ultimately disrupting blood–brain barrier (BBB) tight junction proteins and increasing cerebral microvascular permeability (34, 35). Recent studies have further confirmed that TyG-related metabolic dysfunction significantly impairs BBB integrity and is associated with unfavorable stroke prognosis (36). Under conditions of mild or single-vessel ICAS, preserved perfusion reserve and endothelial repair capacity can partially offset such metabolic damage. However, severe and multi-vessel ICAS leads to persistent hypoperfusion, endothelial dysfunction, and exhausted collateral compensation, leaving the brain extremely vulnerable to superimposed metabolic insults.

Furthermore, TyG-driven insulin resistance activates a systemic pro-inflammatory and prothrombotic state characterized by elevated plasminogen activator inhibitor-1 (PAI-1) activity, platelet hyperaggregability, and fibrinolytic resistance, promoting plaque instability and distal microembolization (37–40). When accompanied by hemodynamic disturbances from ICAS, these metabolic and thrombotic abnormalities exacerbate cerebral ischemic injury. In addition, insulin resistance suppresses endothelial nitric oxide synthase (eNOS) activity and reduces nitric oxide bioavailability, resulting in disrupted neurovascular coupling and impaired vasodilatory reserve (41–44). When neurovascular regulatory dysfunction coincides with exhausted cerebrovascular perfusion reserve in severe ICAS patients, neuronal metabolic demands cannot be adequately compensated, leading to excitotoxic injury and progressive neuronal loss.

This study has several limitations. First, the TyG index was measured only at admission, and serial follow-up measurements were not available, which prevents us from evaluating its longitudinal association with stroke outcomes. Second, the study was limited to Chinese patients recruited from a single center; therefore, the generalizability of our findings to other populations is limited. Third, the cross-sectional nature of our baseline assessment means we cannot fully rule out residual confounders (e.g., C-reactive protein, neutrophil-to-lymphocyte ratio), which may modify the observed predictive effect of the TyG index. Fourth, insulin levels were not routinely tested in our clinical practice due to cost constraints, so we were unable to directly compare multiple insulin resistance indicators and their associations with poor functional outcomes after ischemic stroke. This topic deserves further investigation in larger clinical or community cohorts. Fifth, DSA was not routinely performed for intracranial vascular evaluation in our center. Although CTA/MRA has good concordance with DSA, we cannot achieve gold-standard vascular evaluation. Large-scale prospective studies are needed to validate our overall findings.

## 5 Conclusion

In summary, an elevated TyG index combined with ICAS burden could serve as a valid biological marker that can be used to identify patients at high risk of poor stroke prognosis, even after controlling for established risk variables. However, further research is needed to fully elucidate the mechanisms underlying this association and to validate these findings in diverse populations.

## Data Availability

The original contributions presented in the study are included in the article/supplementary material. Further inquiries can be directed to the corresponding authors.
